# Tissue-specific changes in endophytic bacterial and fungal communities of two pine species associated with pine wilt disease

**DOI:** 10.3389/fmicb.2026.1864084

**Published:** 2026-06-15

**Authors:** Yuan Xu, Ruifen Huang, Hao Yang, Xin Li, Shasha Zhang, Juan Shi

**Affiliations:** 1 Beijing Key Laboratory for Forest Pest Control, Beijing Forestry University, Beijing, China; 2 Hebei Xiong'an New Area City Ecosystem Observation and Research Station, Xiong'an, China; 3 National Forestry and Grassland Administration Center of Prevention and Control Biological Disaster, Shenyang, China; 4 Yanting County Bureau of Natural Resources, Yanting, China

**Keywords:** 16S rRNA, community differentiation, endophytic microbiome, ITS, pine wilt disease, tissue specificity

## Abstract

Pine wilt disease severely threatens pine forests worldwide, yet coordinated shifts in endophytic bacterial and fungal communities across host tissues remain incompletely resolved. We analyzed 16S rRNA gene and ITS amplicons from needles, stems, and roots of healthy trees and naturally infected trees at the mid-to-late stages of disease in *Pinus densiflora* Siebold & Zucc. (Japanese red pine) and *Pinus thunbergii* Parl. (Japanese black pine). Tissue-stratified analyses revealed significant disease-associated differentiation of both bacterial and fungal communities in all three tissues. Bacterial communities showed stronger disease-associated restructuring, with significant shifts in both composition and dispersion, indicating centroid changes accompanied by increased within-group heterogeneity. Fungal communities also differed significantly with disease status, but dispersion changes were not detected. Needles harbored the greatest numbers of differential taxa in both bacterial and fungal communities, whereas fungal community-level differentiation was strongest in stems. Host species background significantly modulated needle bacterial communities as well as needle- and stem-associated fungal communities. Overall, pine wilt disease was associated with tissue-specific reorganization of the endophytic microbiome, with bacterial communities exhibiting greater heterogeneity and fungal communities showing compositional differentiation without detectable dispersion shifts.

## Introduction

Pine wilt disease (PWD), caused by the pinewood nematode *Bursaphelenchus xylophilus*, is among the most destructive diseases affecting pine forest ecosystems worldwide (Back et al., 2024; Silva et al., 2026). After invasion, the nematode disrupts host vascular function and water transport and accelerates physiological decline, ultimately leading to rapid wilting and death (Mamiya, 1983; Back et al., 2024). Studies of PWD have long focused on nematode pathogenicity, vector-mediated transmission, and host physiological responses, whereas the roles and dynamics of host-associated microbial communities within the broader PWD complex remain comparatively understudied (Alves et al., 2018; Leveau, 2024; Li et al., 2024).

Recent plant microbiome research has highlighted that plant health depends not only on the host itself but also on the composition, assembly, and stability of its associated microbiota (Fitzpatrick et al., 2020; Leveau, 2024). Endophytic bacteria and fungi are widely distributed in roots, stems, and leaves and can contribute to nutrient acquisition, stress tolerance, immune modulation, and pathogen suppression (Hardoim et al., 2015; Santoyo et al., 2016; Carrión et al., 2019). Under pathogen invasion or environmental stress, endophytic communities may be reorganized, and these changes may both reflect host imbalance and, in some contexts, feed back on disease outcomes through dysbiosis or altered protective functions (Agler et al., 2016; Carrión et al., 2019; Pfeilmeier et al., 2024). A microbiome-based perspective may therefore complement current ecological explanations of PWD by clarifying disease-associated internal community shifts across host tissues and contexts.

Previous studies have shown that bacterial and fungal communities in PWD-associated samples often differ markedly from those in healthy trees (Proença et al., 2017; Alves et al., 2018; Vicente et al., 2022; Hao et al., 2022; Li et al., 2024; Cao et al., 2024; Vicente et al., 2025). However, three major gaps remain. First, many studies have focused on a single microbial kingdom or a single component of the PWD complex, whereas fungal communities and multi-kingdom patterns have received less systematic attention (Vicente et al., 2022; Cao et al., 2024). Second, needles, stems, and roots represent distinct microenvironments with different ecological filters, but whether disease-associated microbiome shifts show consistent tissue specificity across host species remains unclear (Hao et al., 2022; Li et al., 2024). Third, it is still unresolved whether divergence between healthy and naturally infected trees mainly reflects stable taxon replacement or is also accompanied by strong expansion of within-group heterogeneity, particularly when bacterial and fungal responses are considered together (Proença et al., 2017; Cao et al., 2024).

To address these questions, this study characterized endophytic bacterial and fungal communities in needles, stems, and roots of healthy trees and naturally infected mid-to-late-stage trees of *Pinus densiflora* Siebold & Zucc. (Japanese red pine) and *Pinus thunbergii* Parl. (Japanese black pine). Specifically, asked whether endophytic bacterial and fungal communities differ between healthy and naturally infected trees across tissues, and also if the two microbial kingdoms respond in similar ways, and if these responses would show a clear tissue specificity and host species dependence. The results provide a basis for understanding disease-associated microbiome dynamics and for guiding future screening of functionally relevant microbes.

## Materials and methods

### Sample collection, disease diagnosis, and experimental design

Samples were collected from a mixed plantation of Japanese black pine (*Pinus thunbergii* Parl.) and Japanese red pine (*Pinus densiflora* Siebold & Zucc.) in Weihai, Shandong Province, China, where environmental conditions were broadly comparable among sampled trees. Three healthy and three naturally infected trees of each species were selected, with approximately 10 m between neighboring trees. Healthy trees had fully green needles, normal resin secretion, and no detectable pine wood nematodes. The naturally infected trees represented the mid-to-late stage of disease development and consisted of current-year dead trees with red-brown needles that had not yet abscised, visible insect feeding scars on the bark, and cessation of resin secretion.

Needles, stem tissue at breast height, and lateral roots from 5–20 cm below the soil surface were collected from each tree. A subset of samples was transported to the laboratory at low temperature under sterile conditions for amplicon sequencing, whereas additional woody samples were used for pine wood nematode detection. Nematodes were isolated using the Baermann funnel method. Only dead wood with more than 100 nematodes per gram was selected to represent naturally infected trees at the mid-to-late stage of disease, whereas healthy samples were nematode-free. Nematode identity was confirmed by species-specific PCR using the primer pair P1 (5′-CTACGTGCTGTTGTTGAGTTGGC-3′) and P2 (5′-TGGTGCCTAACATTGCGCGA-3′), corresponding to the P155/P538 primers originally reported by Wang et al. (2009). The design included three biological replicates for each combination of pine species, infection state, and tissue, resulting in 36 samples in the 16S dataset and 36 samples in the ITS dataset.

### Surface sterilization, DNA extraction, PCR amplification, and sequencing

Plant samples were first rinsed with distilled water to remove surface debris and then surface-sterilized sequentially in sterile water for 30 s, 75% ethanol for 1 min, 2% NaClO for 3 min, 75% ethanol for 1 min, and sterile water for 3 min. Stem and root tissues were cut into approximately 5 mm × 5 mm pieces, whereas needles were cut into approximately 10 mm × 10 mm segments and stored at −80 °C until DNA extraction. To verify the effectiveness of surface sterilization, the final rinse water was plated on NA and PDA media and also used as the template for PCR amplification with 799F/1193R and ITS1F/ITS2R. No bacterial or fungal colonies were recovered from the final rinse water, and agarose gel electrophoresis showed no detectable PCR bands, indicating that surface-associated microorganisms had been effectively removed.

Total microbial DNA was extracted using the MagAttract PowerSoil Pro DNA Kit (Qiagen). DNA quality was checked by 1% agarose gel electrophoresis and preliminarily quantified using a NanoDrop 2000 spectrophotometer. For bacterial community profiling, the V5-V7 region of the 16S rRNA gene was amplified with primers 799F/1193R (Beckers et al., 2016). For fungal community profiling, the ITS region was amplified with primers ITS1F/ITS2R (Gardes and Bruns, 1993; Op De Beeck et al., 2014). The 16S PCR mixture (20 μL) contained 8.0 μL RNase-free water, 0.4 μL DNA template, 0.8 μL of each primer, and 10.0 μL 2 × Pro Taq. Thermal cycling consisted of 94 °C for 3 min, followed by 27 cycles of 94 °C for 30 s, 55 °C for 30 s, and 72 °C for 45 s, with a final extension at 72 °C for 10 min. The ITS PCR mixture (24 μL) contained 12.0 μL RNase-free water, 0.4 μL DNA template, 0.8 μL of each primer, and 10.0 μL 2 × Pro Taq. Thermal cycling consisted of 94 °C for 3 min, followed by 35 cycles of 94 °C for 30 s, 55 °C for 30 s, and 72 °C for 45 s, with a final extension at 72 °C for 10 min.

PCR products were purified using a PCR Clean-Up Kit, quantified with a Qubit 4.0 fluorometer, pooled at equimolar concentrations, and sequenced by Shanghai Majorbio Bio-Pharm Technology Co., Ltd. (Shanghai, China) on an Illumina MiSeq platform (2 × 300 bp). Raw sequencing data were demultiplexed by the sequencing provider and returned as per-sample paired-end FASTQ files. All downstream bioinformatic analyses reported in this study were performed independently by the authors.

### Processing of 16S data and bacterial community analysis

Provider-delivered paired-end FASTQ files were processed in R 4.4.2 using the dada2 package (Callahan et al., 2016). Forward and reverse reads were quality filtered, denoised, merged, and screened for chimeras to infer amplicon sequence variants (ASVs). ASVs assigned to chloroplasts or mitochondria were removed prior to downstream analyses. Taxonomic assignment was performed against the SILVA nr99 v138.1 database (Quast et al., 2013). ASV-level and genus-level bacterial community objects were constructed in phyloseq (McMurdie and Holmes, 2013). ASV-level data were used for sequencing depth statistics, rarefaction curves, Good’s coverage, alpha diversity, and beta diversity analyses, whereas genus-level data were used for taxonomic composition summaries and differential abundance analysis. All subsequent statistical analyses and figure generation were conducted in R.

### Processing of ITS data and fungal community analysis

Provider-delivered paired-end FASTQ files were processed in R 4.4.2 using the dada2 package (Callahan et al., 2016). Reads were quality filtered, denoised, merged, and screened for chimeras to infer ASVs. Taxonomic assignment was performed against the UNITE dynamic fungi database (version 2025-02-19, no singletons) (Nilsson et al., 2019), and non-fungal ASVs were removed before downstream analyses. ASV-level and genus-level fungal community objects were constructed in phyloseq (McMurdie and Holmes, 2013). ASV-level data were used for sequencing depth statistics, rarefaction curves, Good’s coverage, alpha diversity, and beta diversity analyses, whereas genus-level data were used for taxonomic composition summaries and differential abundance analysis. All subsequent statistical analyses and figure generation were conducted in R.

### Alpha diversity analysis

For both bacterial and fungal datasets, sequencing depth, observed ASV richness, Shannon diversity, and Good’s coverage were calculated. In addition, the dominant genus and its relative abundance were extracted from genus-level relative abundance matrices to assess shifts in community evenness and single-genus dominance across infection states. Summary statistics were reported as medians for each combination of pine species, tissue, and infection state. Within each tissue, Wilcoxon rank-sum tests were used to compare alpha diversity indices between healthy and naturally infected groups. To evaluate host species effects and their interaction with infection state, alpha diversity indices within each tissue were further rank-transformed and analyzed using Condition × Species models as supplementary analyses. When multiple alpha-diversity metrics showed identical sample rank ordering within a tissue, the corresponding rank-based model statistics were also identical by construction.

### Beta diversity and community structure analysis

Bray-Curtis distances were calculated from ASV-level relative abundance matrices, and principal coordinate analysis (PCoA) was used to visualize community-level differences. To avoid pooling all combinations into a single analysis, we adopted a tissue-stratified strategy and compared healthy and naturally infected groups separately within needles, stems, and roots. PERMANOVA was performed with adonis2 using the model Condition + Species + Condition × Species within each tissue and 999 permutations (Anderson, 2001). Betadisper was further used to test whether healthy and naturally infected groups differed in within-group dispersion, and dispersion significance was evaluated with 999-permutation tests, thereby distinguishing centroid shifts from increases in within-group heterogeneity (Anderson, 2006). Because each Species × Condition × Tissue combination included only three biological replicates, the Species and interaction terms in these multivariable tests were interpreted cautiously and used primarily to describe effect patterns rather than to make fine-grained population-level claims.

### Differential taxa analysis

Differential abundance analysis was performed at the genus level using ANCOM-BC (Lin and Peddada, 2020). Infection state was specified as the main explanatory variable and pine species was included as a covariate. The model was defined as Condition + Species, and taxa with *Q* < 0.05 were considered significant. Representative differential taxa were identified separately for needles, stems, and roots, and the sign of the log fold change was used to determine whether a taxon was enriched in naturally infected or healthy samples.

### Statistical analysis and visualization

All downstream statistical analyses and visualizations were performed in R 4.4.2 using dada2, ShortRead, phyloseq, vegan, dplyr, ggplot2, and ANCOMBC. Rarefaction curves and Good’s coverage were calculated at the ASV level. Mean relative abundances were summarized at the phylum and genus levels for community composition plots, and shared and unique ASV and genus-level taxa were counted by tissue.

## Results

### Sequencing overview and overall characteristics of bacterial and fungal communities

The study included 36 bacterial 16S samples and 36 fungal ITS samples. Read-tracking results showed that the 16S dataset contained 62,020–123,738 raw reads per sample and a median post-chimera retention rate of 92.21%, whereas the ITS dataset contained 45,741–120,592 raw reads per sample and a median post-chimera retention rate of 96.68% (Supplementary Table S1). In the final community objects, the 16S dataset retained 11,632 ASVs and 1,099 genus-level taxa, with sequencing depths ranging from 54,648 to 108,171 reads, whereas the ITS dataset retained 1,243 ASVs and 286 genus-level taxa, with sequencing depths ranging from 43,232 to 116,080 reads (Table 1; Supplementary Table S1). Overall, bacterial communities showed greater taxonomic complexity than fungal communities.

**Table 1:** Sequencing and annotation overview of the 16S and ITS datasets.

Dataset	Samples	ASVs	Genera	MinDepth	MaxDepth
16S	36	11,632	1,099	54,648	108,171
ITS	36	1,243	286	43,232	116,080

Rarefaction curves approached saturation in both datasets, and Good’s coverage values were close to 1.0 (Supplementary Figures S5, S6; Supplementary Table S1). At the phylum level, bacterial communities were generally dominated by *Proteobacteria*, whereas naturally infected needles showed increased relative abundances of *Actinobacteriota*, *Firmicutes*, and *Bacteroidota*. Fungal communities were mainly composed of *Ascomycota* and *Basidiomycota*, with a higher proportion of *Ascomycota* in naturally infected needles and relatively higher *Basidiomycota* abundance in healthy stems (Supplementary Figures S1–S4; Supplementary Table S6).

Shared and unique taxon statistics further highlighted strong tissue differentiation. In the 16S dataset, 104 ASVs were shared among all three tissues, whereas needles contained 8,001 tissue-specific ASVs. In the ITS dataset, only 11 ASVs were shared among all three tissues, whereas needles contained 739 unique ASVs. When only naturally infected samples were considered, needles still accounted for 7,993 unique bacterial ASVs and 549 unique fungal ASVs (Supplementary Table S5).

Dominant genus statistics also showed contrasting organizational patterns between the two microbial kingdoms. In the 16S dataset, *Achromobacter* was the dominant genus in 24 of 36 samples, whereas in the ITS dataset, *Yamadazyma* was dominant in 8 of 36 samples. Thus, strong single-genus dominance was more common in bacterial communities than in fungal communities.

### Endophytic bacterial communities differed significantly between healthy and naturally infected trees and showed increased heterogeneity

**Figure 1:**
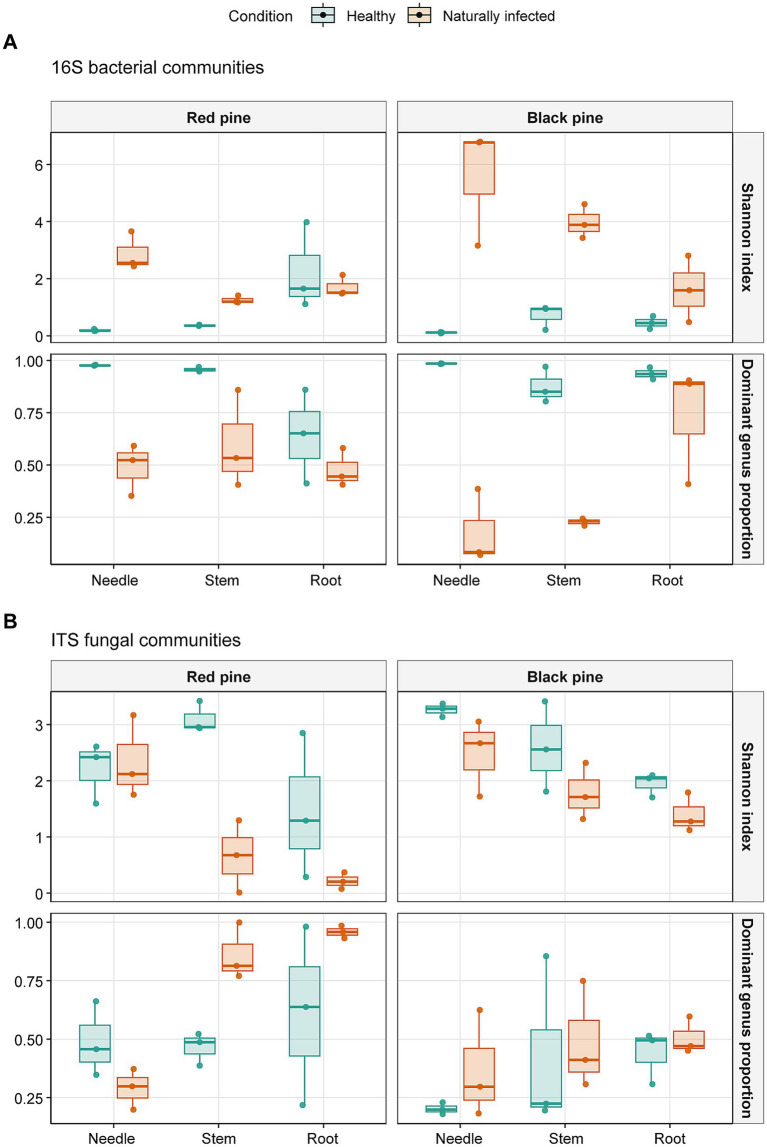
Alpha diversity and dominance patterns of endophytic bacterial and fungal communities in healthy and naturally infected pine trees across tissues. Panel **(A)** shows bacterial 16S communities and panel **(B)** shows fungal ITS communities. Shannon diversity and the relative abundance of the dominant genus in each sample are presented to compare shifts in community evenness under infection-associated stress.

Alpha diversity analysis of the 16S dataset showed that bacterial community shifts were most pronounced in needles and stems (Figure 1A; Supplementary Table S2). In needles, observed ASV richness, Shannon diversity, and dominant genus proportion all differed significantly between healthy and naturally infected trees (all *p* = 0.002). In stems, Shannon diversity and dominant genus proportion also changed significantly (*p* = 0.002 and 0.009, respectively), whereas none of the alpha diversity metrics differed significantly in roots. For example, in black pine needles, the median Shannon index increased from 0.11 in healthy trees to 6.77 in naturally infected trees, and in red pine needles it increased from 0.18 to 2.55. Healthy samples generally showed a high dominant-genus proportion, whereas naturally infected samples showed a lower level of single-genus dominance. Supplementary rank-based models further showed significant Condition × Species interactions for needle bacterial Shannon diversity and dominant-genus proportion (Supplementary Table S2), indicating that pooled needle alpha patterns were moderated by host species background.

**Figure 2:**
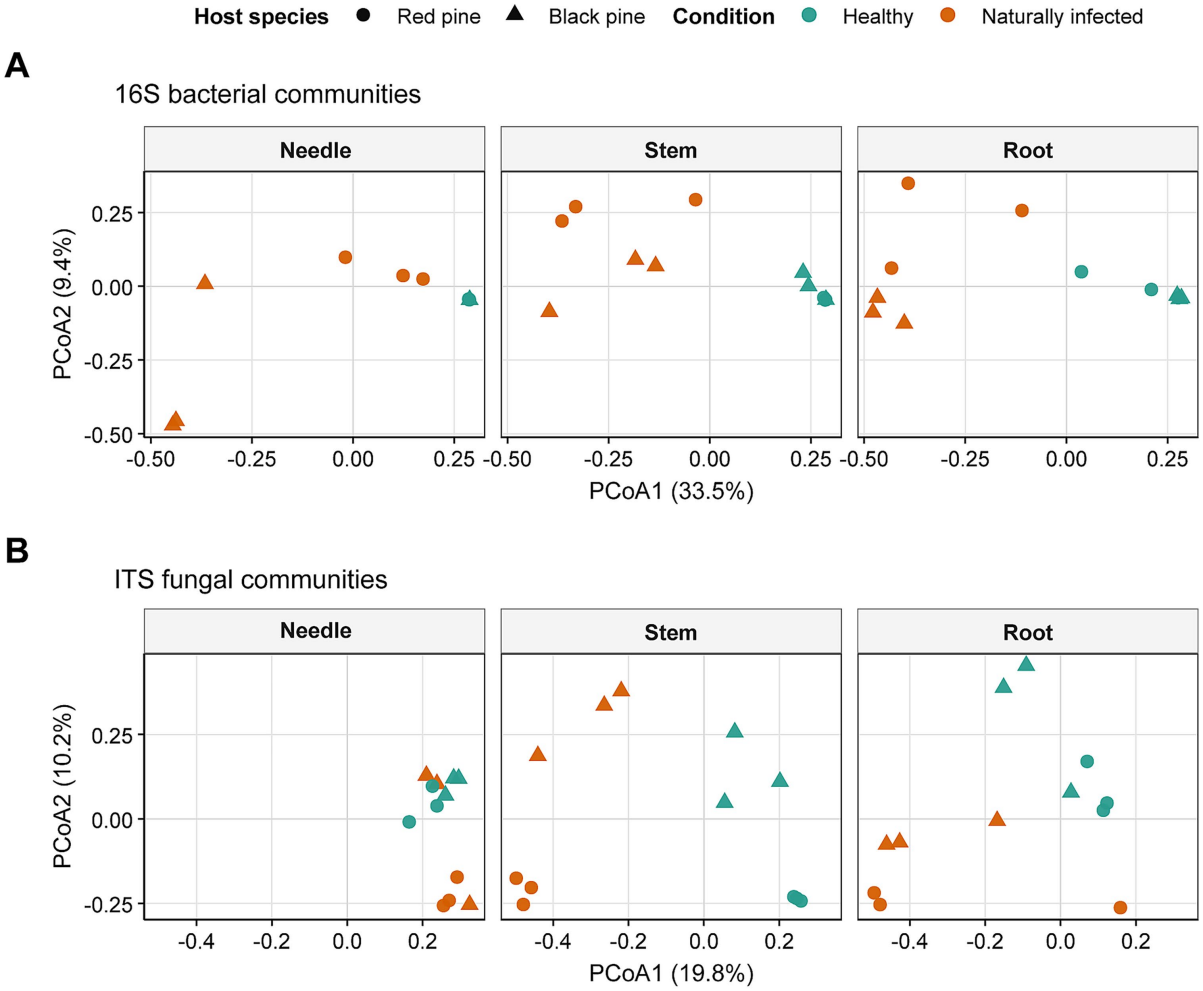
Principal coordinate analysis of ASV-level Bray-Curtis distances for the 16S and ITS datasets. Panel **(A)** shows bacterial 16S communities and panel **(B)** shows fungal ITS communities. Colors indicate health status, symbols indicate pine species, and facets indicate tissue type. The plot illustrates the overall differentiation of bacterial and fungal community structures between healthy and naturally infected trees.

PCoA showed clear separation between healthy and naturally infected samples in needles, stems, and roots at the bacterial community level (Figure 2A). Tissue-stratified PERMANOVA identified infection state as a significant explanatory factor in all three tissues, accounting for 33.28, 36.32, and 35.68% of the variance in needles, stems, and roots, respectively (Needle: *R*^2^ = 0.333, *p* = 0.001; Stem: *R*^2^ = 0.363, *p* = 0.001; Root: *R*^2^ = 0.357, *p* = 0.001; Table 2; Supplementary Table S3). Betadisper permutation tests were also significant in all three tissues (Needle: *p* = 0.001; Stem: *p* = 0.001; Root: *p* = 0.001; Table 2; Supplementary Table S3), and naturally infected samples consistently showed greater mean within-group distances than healthy samples. For bacterial communities, significant PERMANOVA together with significant betadisper indicates that disease-associated differentiation reflected both centroid shifts and increased within-group heterogeneity.

**Table 2:** Comparison of community differentiation and explanatory power in the 16S and ITS datasets.

Dataset	Global			Needle			Stem			Root			Significant taxa		
	*R* ^2^	*P*	Disp. P	*R* ^2^	*P*	Disp. P	*R* ^2^	*P*	Disp. P	*R* ^2^	*P*	Disp. P	Needle	Stem	Root
16S	0.262	0.001	0.001	0.333	0.001	0.001	0.363	0.001	0.001	0.357	0.001	0.001	129	43	53
ITS	0.093	0.001	0.072	0.166	0.007	0.983	0.316	0.001	0.243	0.212	0.001	0.108	76	30	13

*R*^2^ = coefficient of determination; P = PERMANOVA *p*-value; Disp. P = betadisper *p*-value (dispersion test). Significant Taxa = number of differentially abundant taxa detected by ANCOM-BC (*Q* < 0.05).

**Figure 3:**
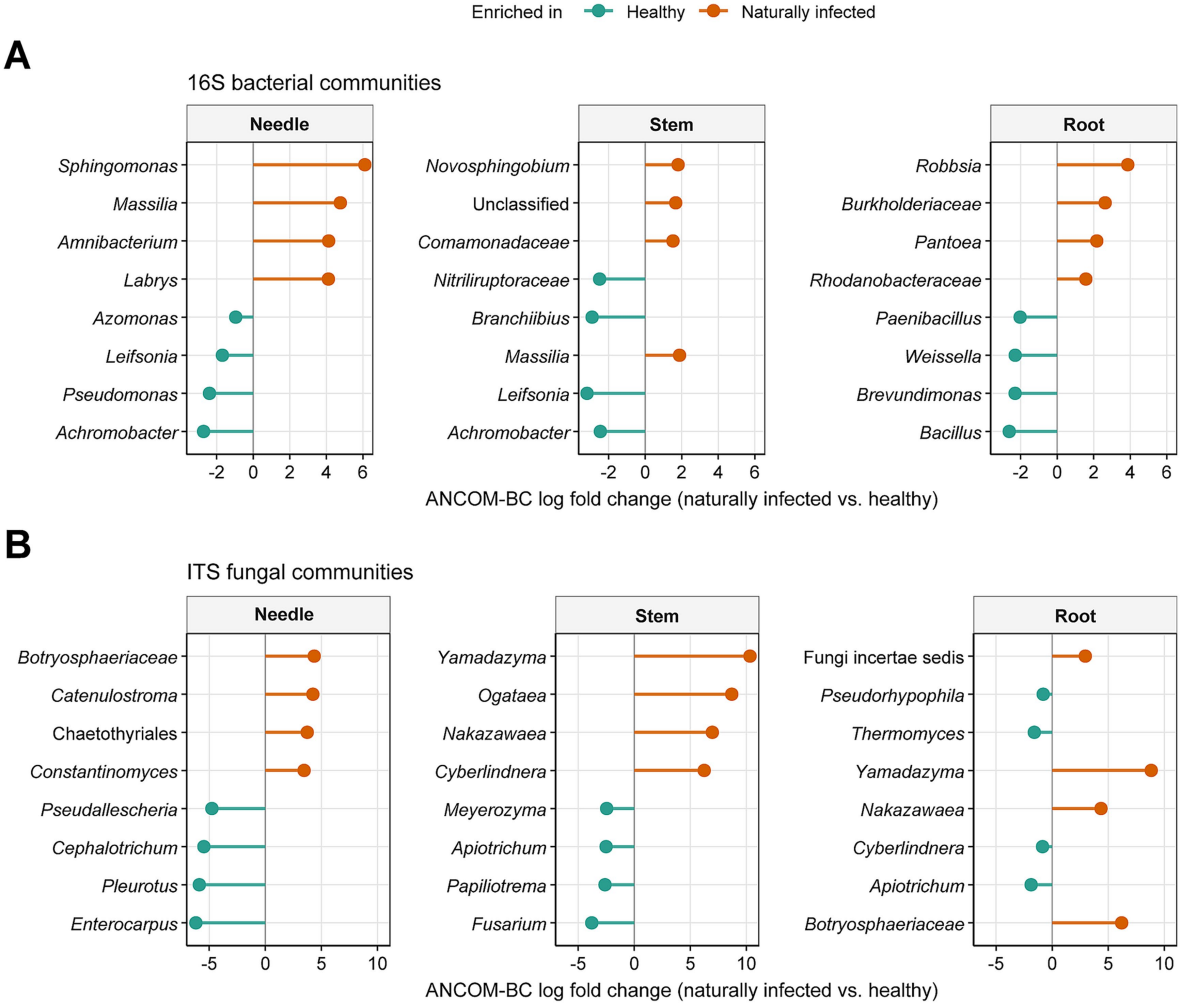
Representative differential taxa across tissues in the 16S and ITS datasets. Panel **(A)** shows bacterial 16S communities and panel **(B)** shows fungal ITS communities. The x-axis shows the ANCOM-BC log fold change (naturally infected vs. healthy); positive values indicate enrichment in naturally infected samples and negative values indicate enrichment in healthy samples. The figure highlights the major replacement directions and tissue-specific responses of bacterial and fungal communities.

Differential abundance analysis detected 129, 43, and 53 significant bacterial taxa in needles, stems, and roots, respectively (Figure 3A; Table 2; Supplementary Table S4), with the largest response occurring in needles. In needles, naturally infected samples were enriched in *Sphingomonas*, *Massilia*, *Amnibacterium*, and *Labrys*, whereas healthy samples were enriched in *Achromobacter*, *Pseudomonas*, and *Leifsonia*. In stems, naturally infected samples were enriched in *Massilia*, *Novosphingobium*, and *Comamonadaceae*-related taxa, whereas healthy samples were enriched in *Leifsonia*, *Branchiibius*, *Nitriliruptoraceae*, and *Achromobacter*. In roots, naturally infected samples were enriched in *Robbsia*, *Burkholderiaceae*, *Pantoea*, and *Rhodanobacteraceae*-related taxa, whereas healthy samples were enriched in *Bacillus*, *Brevundimonas*, *Weissella*, and *Paenibacillus*. These patterns indicate marked tissue specificity in bacterial community change associated with infection state.

### Endophytic fungal communities also differed significantly between healthy and naturally infected trees and showed tissue-specific replacement

Alpha diversity analysis of the ITS dataset showed tissue-specific fungal responses to natural infection (Figure 1B; Supplementary Table S2). In needles, observed ASV richness differed significantly between healthy and naturally infected trees (*p* = 0.026), whereas Shannon diversity and the relative abundance of the dominant genus did not. In stems, both observed ASV richness and Shannon diversity were significantly lower in naturally infected trees than in healthy trees (*p* = 0.002 and 0.004, respectively). In roots, Shannon diversity showed only a marginal difference in the Wilcoxon comparison (*p* = 0.065); however, the supplementary rank-based model identified a significant effect of Condition after accounting for host species (Supplementary Table S2). Consistent with this pattern, healthy black pine needles and stems showed relatively high fungal diversity, whereas naturally infected red pine stems and roots exhibited lower Shannon diversity, with median values of 0.68 and 0.20, respectively. The supplementary rank-based models also revealed a significant Condition × Species interaction for stem Shannon diversity (Supplementary Table S2), indicating that the effect of infection condition on stem fungal alpha diversity depended on host species.

PCoA also showed clear separation between healthy and naturally infected groups in the ITS dataset (Figure 2B). Tissue-stratified PERMANOVA indicated that infection state was significantly associated with fungal community structure in needles, stems, and roots, explaining 16.58, 31.57, and 21.23% of the variance, respectively (Needle: *R*^2^ = 0.166, *p* = 0.007; Stem: *R*^2^ = 0.316, *p* = 0.001; Root: *R*^2^ = 0.212, *p* = 0.001; Table 2; Supplementary Table S3). The strongest response occurred in stems. In contrast to the 16S dataset, none of the three tissues showed significant between-group dispersion shifts in betadisper permutation tests (Needle: *p* = 0.983; Stem: *p* = 0.243; Root: *p* = 0.108; Table 2; Supplementary Table S3), indicating that fungal community change mainly reflected between-group structural separation rather than a strong increase in within-group heterogeneity.

ANCOM-BC identified 76, 30, and 13 significant fungal taxa in needles, stems, and roots, respectively (Figure 3B; Table 2; Supplementary Table S4), with needles again showing the largest number of differential taxa. In needles, naturally infected samples were enriched in *Botryosphaeriaceae*, *Catenulostroma*, *Chaetothyriales*-related taxa, and *Constantinomyces*, whereas healthy samples were enriched in *Enterocarpus*, *Pleurotus*, *Cephalotrichum*, and *Pseudallescheria*. In stems, naturally infected samples were enriched in *Yamadazyma*, *Ogataea*, and *Nakazawaea*, whereas healthy samples were enriched in *Fusarium*, *Papiliotrema*, and *Apiotrichum*. In roots, naturally infected samples were enriched in *Yamadazyma* and *Botryosphaeriaceae*, whereas healthy samples were enriched in *Apiotrichum*, *Thermomyces*, and *Cyberlindnera*. These results likewise support strong tissue specificity in fungal community change associated with infection state.

### Host species background modulated infection-associated differences in selected tissues

Tissue-stratified PERMANOVA showed that host species background significantly affected selected tissues. In the 16S dataset, both the Species term and the Condition × Species interaction were significant for needle bacterial communities (*R*^2^ = 0.1568 and 0.1565, both *p* = 0.017), whereas species effects in stems and roots were weak or non-significant (Supplementary Table S3). In the ITS dataset, both the Species term and the interaction were significant in needles (*R*^2^ = 0.130 and 0.129, *p* = 0.029 and 0.034), and species effects were even stronger in stems (*R*^2^ = 0.197 and 0.170, *p* = 0.002 and 0.003), whereas no significant interaction was detected in roots (Supplementary Table S3).

When the two pine species were analyzed separately, Condition remained an important explanatory factor in both bacterial and fungal communities. In the 16S dataset, Condition was significant in both red pine and black pine (both *p* = 0.001). In the ITS dataset, Condition also remained significant in both species (red pine: *p* = 0.001; black pine: *p* = 0.011; Supplementary Table S3). These results indicate that host species background primarily modulated the magnitude and pattern of infection-associated change in needle bacteria and in needle and stem fungi, rather than replacing infection state as the main explanatory axis.

### Bacterial and fungal communities showed distinct response patterns across infection states

When the 16S and ITS datasets were considered together, both bacterial and fungal communities differed significantly between healthy and naturally infected trees across all three tissues. Bacterial communities consistently showed higher explanatory power than fungal communities and were the only group to show significant betadisper differences in all tissues. By contrast, fungal communities also showed significant between-group differentiation across tissues, but none of the dispersion tests reached significance. The taxa contributing to these shifts also differed between the two microbial kingdoms. Bacterial change mainly involved *Sphingomonas*, *Massilia*, *Novosphingobium*, *Achromobacter*, and *Burkholderiaceae-* and *Comamonadaceae-*related groups, whereas fungal change mainly involved *Yamadazyma*, *Ogataea*, *Nakazawaea*, and *Botryosphaeriaceae*-related taxa. Overall, bacterial communities showed more heterogeneous restructuring associated with infection state, whereas fungal communities showed relatively stable taxon replacement.

## Discussion

### Endophytic bacterial and fungal communities both shifted in association with pine wood nematode infection

Our results demonstrate that endophytic bacterial and fungal communities in needles, stems, and roots differed significantly between healthy trees and naturally infected mid-to-late-stage trees, indicating that pine wilt disease status was associated with microbiome reorganization across multiple host tissues and both microbial kingdoms. This finding aligns with recent PWD microbiome studies in various *Pinus* species, but extends them by simultaneously characterizing bacteria and fungi across three internal tissues within a single framework (Hao et al., 2022; Li et al., 2024; Cao et al., 2024; Vicente et al., 2025). Because our study compared healthy and naturally infected trees at a single time point rather than tracking the same individuals longitudinally, the observed differences are best interpreted as disease-associated restructuring rather than direct temporal responses to initial infection. In the broader context of plant pathology, these results support the view that host-associated microbiota are integral to disease outcomes, with microbial reorganization often accompanying loss of host control and tissue deterioration (Leveau, 2024; Pfeilmeier et al., 2024). Notably, the stronger changes detected in needles and stems suggest that aboveground internal tissues may serve as particularly sensitive indicators of host decline during pine wilt disease (Jiang et al., 2023).

### Bacterial and fungal communities followed different response trajectories

Bacterial and fungal communities did not respond in the same way. In all three tissues, bacterial community differentiation explained more variation than fungal differentiation, and only the bacterial dataset showed significant increases in within-group dispersion. We interpret this contrast as evidence that pine wilt disease creates spatially patchy bacterial niches rather than a single uniform diseased state. PWD is known to disrupt hydraulic continuity, cause cavitation around resin canals, and drive irreversible xylem conduit dysfunction, thereby generating uneven patterns of water loss, solute concentration, host cell damage, and likely oxygen diffusion within woody tissues (Mamiya, 1983; Yazaki et al., 2018). Under such conditions, fast-turnover bacterial assemblages may respond idiosyncratically to local pulses of leaked substrates, oxidative imbalance, resin-derived compounds, and secondary colonization opportunities, which would reduce single-genus dominance and inflate sample-to-sample dispersion. Fungal assemblages, by contrast, may be more strongly constrained by prior colonization, tissue filtering, and persistence within woody microhabitats, so disease progression yields a more repeatable replacement of taxa rather than a generalized expansion of variance. In this framework, higher bacterial betadisper values are consistent not only with dysbiosis, but also with multiple ecological trajectories emerging during late-stage host collapse, whereas the fungal response is more consistent with deterministic turnover within a narrower set of woody endophytic niches (Leveau, 2024; Pfeilmeier et al., 2024).

### Tissue specificity was a major feature of infection-associated microbiome change

Strong tissue specificity was one of the clearest signals in both datasets. Needles contained the largest numbers of differential taxa and tissue-specific ASVs, showing that they were not merely passive endpoints of systemic infection but distinct microhabitats with their own disease-associated filters. This interpretation is consistent with previous pine wilt disease work on foliar microbial change and with the broader view that internal plant compartments represent discrete ecological niches shaped by local resource regimes and host physiology (Jiang et al., 2023; Arnold et al., 2025). The pronounced fungal response in stems was also biologically plausible because pine wilt disease directly disrupts xylem function and woody internal tissues, potentially altering oxygen status, moisture, and available carbon in ways that favor fungal replacement (Mamiya, 1983; Vicente et al., 2012; Vicente et al., 2025). Root responses were more moderate at the community level but remained compositionally distinct, indicating that belowground tissues were connected to disease development rather than insulated from it. In forest systems, root endophytic fungi can covary strongly with root- and soil-associated microbiome structure, supporting the view that root-associated responses may follow their own assembly rules rather than simply mirror aboveground change (Netherway et al., 2024).

### Host species background modulated infection-associated community differences in selected tissues

Although infection state and tissue specificity were the dominant signals in this study, host species background should not be overlooked. Tissue-stratified PERMANOVA detected significant species effects or Condition × Species interactions in needle bacteria as well as needle and stem fungi, whereas roots showed weaker species dependence. Thus, red pine and black pine shared the same broad disease-associated direction of change, but not the same magnitude or configuration of response in every tissue.

This pattern fits broader principles of plant microbiome assembly. Host genotype and host filtering can shape foliar and endophytic communities, and recent work on the internal microbiome of living trees further indicates that woody microbial assemblages can be specialized to host tree species (Bálint et al., 2013; Xiong et al., 2021; Arnold et al., 2025). In our study, species effects were concentrated in aboveground tissues, which is consistent with stronger host filtering where tissue chemistry, defense traits, and internal microclimate are more host dependent.

Within the pine wilt disease literature, most high-throughput studies have focused on a single pine host, making it difficult to separate infection effects from host background effects within a common analytical framework (Hao et al., 2022; Li et al., 2024; Liu et al., 2021; Vicente et al., 2025). By including both *Pinus densiflora* and *P. thunbergii*, the present study shows that infection state remained the primary explanatory factor, while host background still modulated selected tissue responses. This interpretation is also compatible with evidence that different pine hosts or resistant and susceptible materials differ in defense transcription and in pine wood nematode-associated bacterial assemblages after infection (Wang et al., 2023; Xue et al., 2019).

### Candidate taxa associated with disease status

Several differential taxa emerged as plausible indicators of disease status, although these associations should be interpreted cautiously. Among bacteria, *Massilia*, *Sphingomonas*, *Novosphingobium*, and *Burkholderiaceae*-related taxa were repeatedly enriched in naturally infected tissues, consistent with previous pine wilt disease-associated datasets (Hao et al., 2022; Li et al., 2024; Cao et al., 2024). By contrast, *Achromobacter*, *Pseudomonas*, *Bacillus*, and *Paenibacillus* were more characteristic of healthy tissues. Given that some endophytic bacteria, including *Bacillus*, have shown disease-suppressive potential in pine wilt disease systems, these health-associated taxa represent reasonable candidates for future isolation and validation, although relative abundance patterns alone do not establish function (Sun et al., 2024; Ahmed et al., 2025).

On the fungal side, *Yamadazyma*, *Ogataea*, *Nakazawaea*, and *Botryosphaeriaceae*-related taxa were repeatedly associated with naturally infected samples, whereas *Apiotrichum*, *Papiliotrema*, and *Fusarium* were more frequent in healthy tissues (Liu et al., 2021; Vicente et al., 2025). The enrichment of several budding-yeast lineages in diseased stems and roots may reflect adaptation to stressed woody interiors generated during disease progression, but this interpretation remains tentative. Because our ITS data do not directly measure oxygen status, water potential, or absolute fungal biomass, these patterns should be regarded as mechanistic hypotheses rather than causal explanations (Lopes et al., 2015; Garcia-Acero et al., 2024; Araya et al., 2024).

## Limitations and future directions

Several limitations should be considered when interpreting these results. First, our comparison involved healthy trees and naturally infected trees at mid-to-late disease stages, so the observed microbiome shifts likely reflect both pine wilt disease-associated effects and later host decline rather than early infection alone. Second, although surface sterilization appeared effective based on culture and PCR tests of the final rinse water, the extremely high relative abundance of *Achromobacter* in several healthy 16S samples should be interpreted cautiously because extraction blanks, negative controls, and absolute quantification were not included (Callahan et al., 2016; Weiss et al., 2016). Third, because amplicon sequencing data are compositional, enrichment in naturally infected samples cannot be interpreted as absolute proliferation without further validation (Lin and Peddada, 2020). Finally, each Species × Condition × Tissue combination included only three biological replicates, which limits power for multivariable PERMANOVA partitioning and makes dispersion estimates more sensitive to individual samples, particularly for species and interaction terms. Accordingly, our results are best interpreted as evidence of consistent association patterns rather than definitive population-level effect estimates.

Future work should prioritize three directions: targeted isolation and reinoculation of health-associated bacteria and disease-enriched fungi, denser temporal sampling to distinguish early from late microbiome shifts, and integration of amplicon surveys with absolute quantification, metabolomics, and synthetic-community or inoculation experiments. Such approaches will be necessary to determine whether the taxa identified here act as protective endophytes, opportunistic colonizers, or indicators of tissue decline.

## Conclusion

Using 16S rRNA gene and ITS amplicon sequencing, we compared endophytic bacterial and fungal communities in needles, stems, and roots between healthy trees and naturally infected trees at the mid-to-late stages of disease. Disease status was associated with significant bacterial and fungal differentiation in all three tissues, but the two microbial kingdoms showed distinct response patterns. Bacterial communities displayed greater heterogeneity, with significant increases in within-group dispersion, whereas fungal communities mainly showed compositional differentiation without detectable dispersion shifts. Disease-associated microbiome change was strongly tissue-specific: needles harbored the largest numbers of differential taxa in both datasets, whereas stem fungal communities showed the strongest community-level differentiation. Host species background further modulated needle bacterial communities as well as needle and stem fungal communities. Together, these findings show that pine wilt disease is associated with pronounced, tissue-specific reorganization of the endophytic microbiome and provide a foundation for future mechanistic studies and microbial resource discovery.

## Data Availability

The raw sequencing data generated in this study, including 16S rRNA gene and ITS amplicon datasets, have been deposited in the NCBI Sequence Read Archive (SRA) under BioProject accession number PRJNA1469851 (https://www.ncbi.nlm.nih.gov/bioproject/PRJNA1469851). The associated 36 BioSample records are available under accession numbers SAMN60385270–SAMN60385305.
