# Supplementary figures and images for: Tissue-specific changes in endophytic bacterial and fungal communities of two pine species associated with pine wilt disease
Source: Front Microbiol. 2026 Jun 15;17:1864084. doi: 10.3389/fmicb.2026.1864084 (PMC13311027; doi:10.3389/fmicb.2026.1864084)

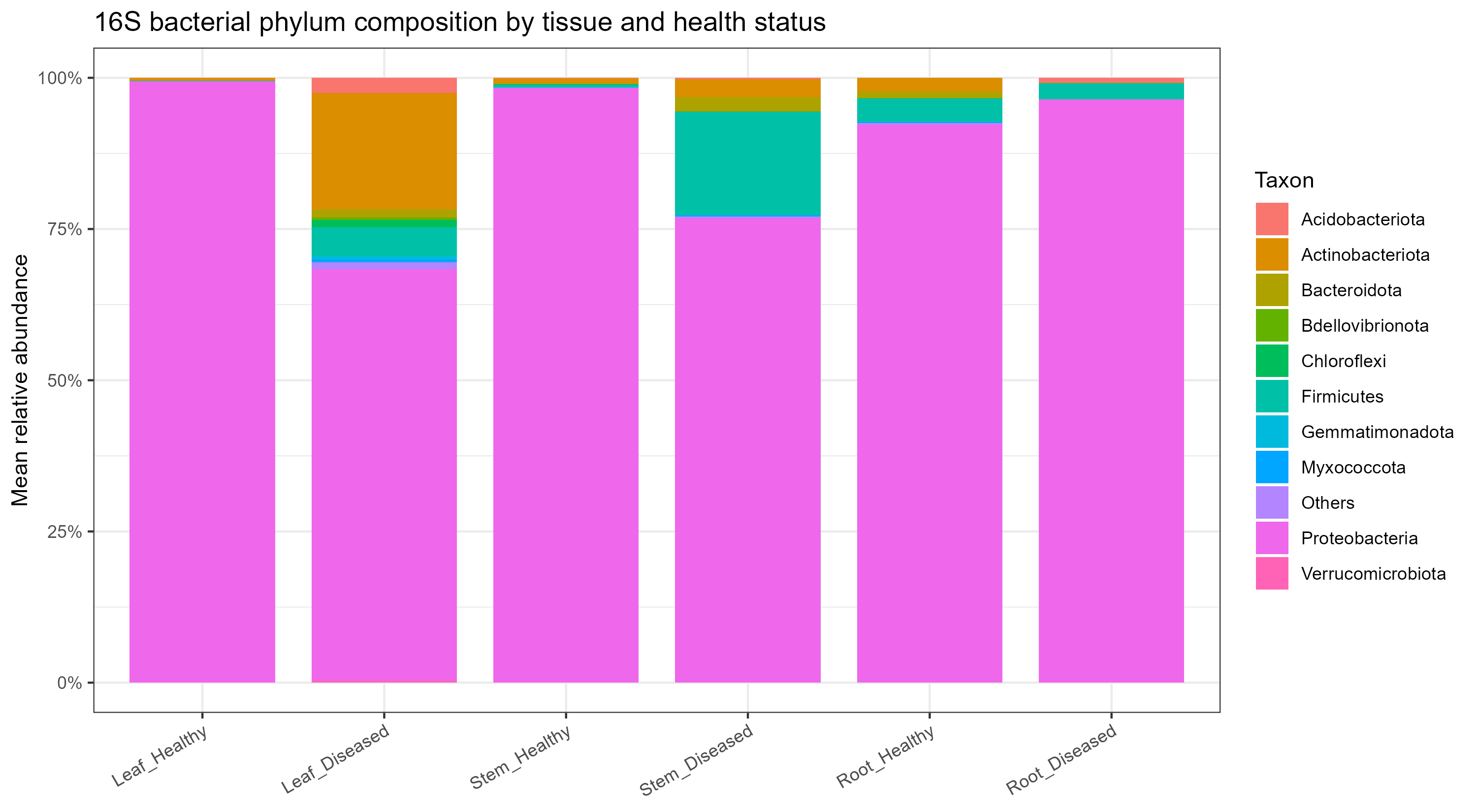

Supplement: Supplementary file 1 [file Image_1.PNG]

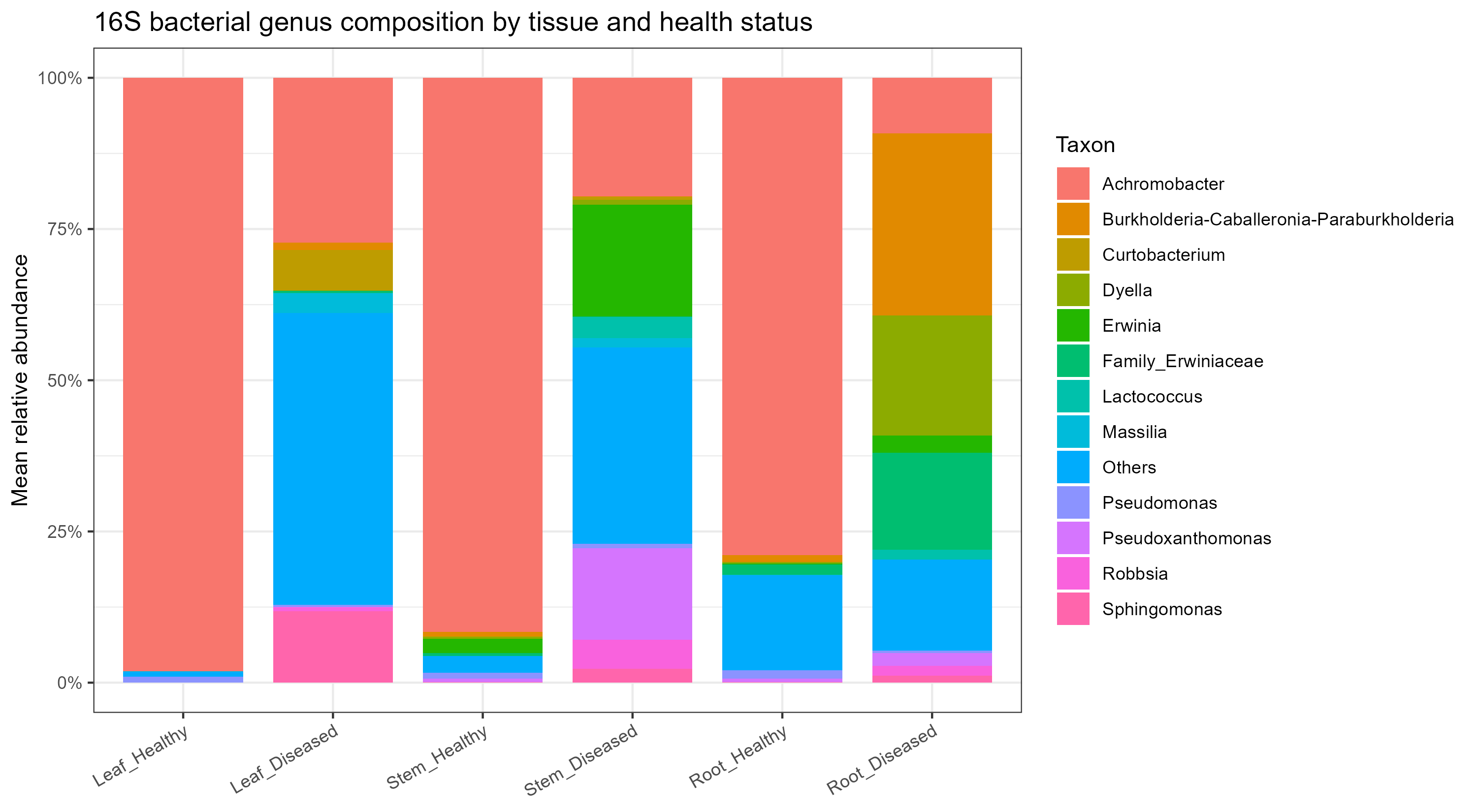

Supplement: Supplementary file 2 [file Image_2.PNG]

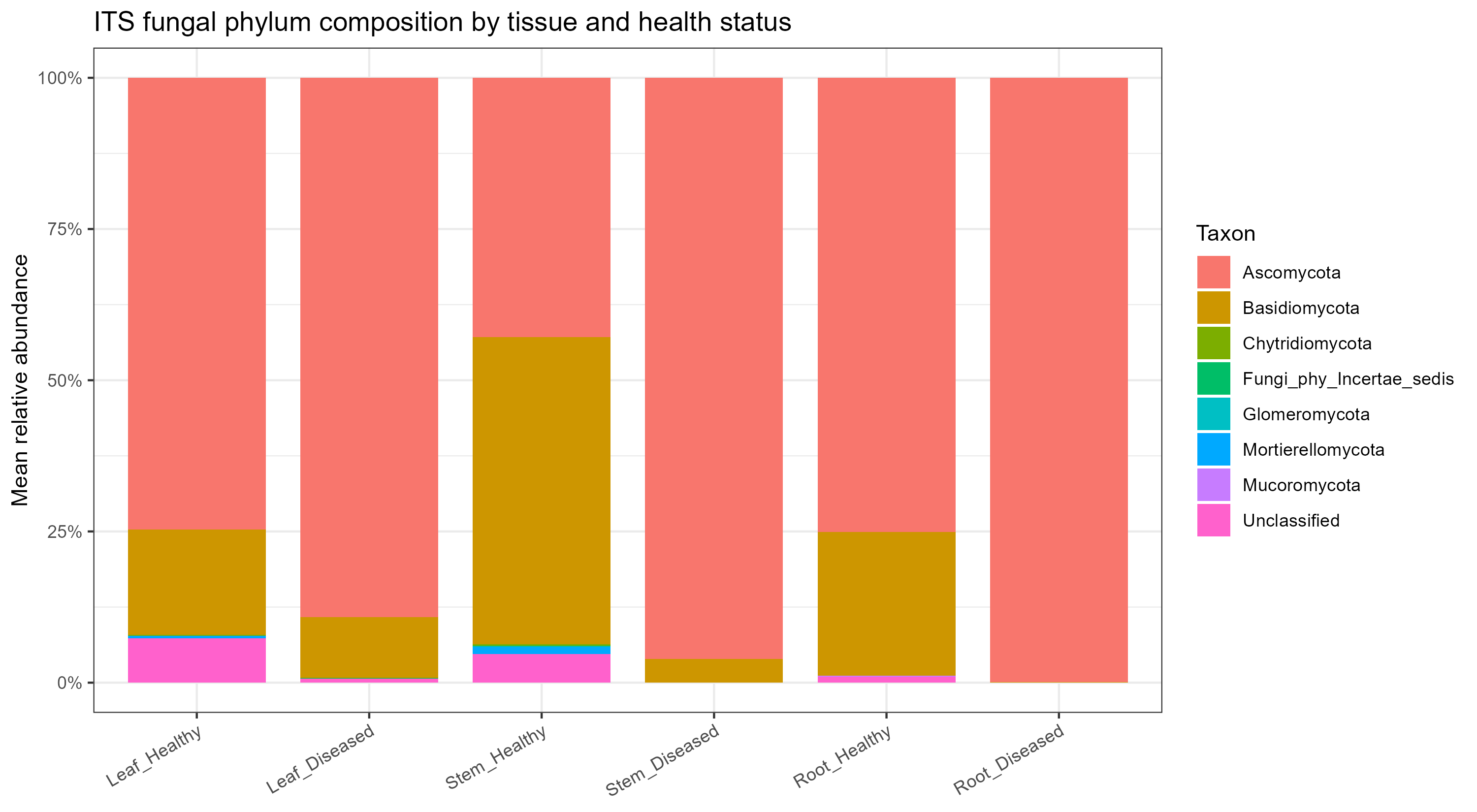

Supplement: Supplementary file 3 [file Image_3.PNG]

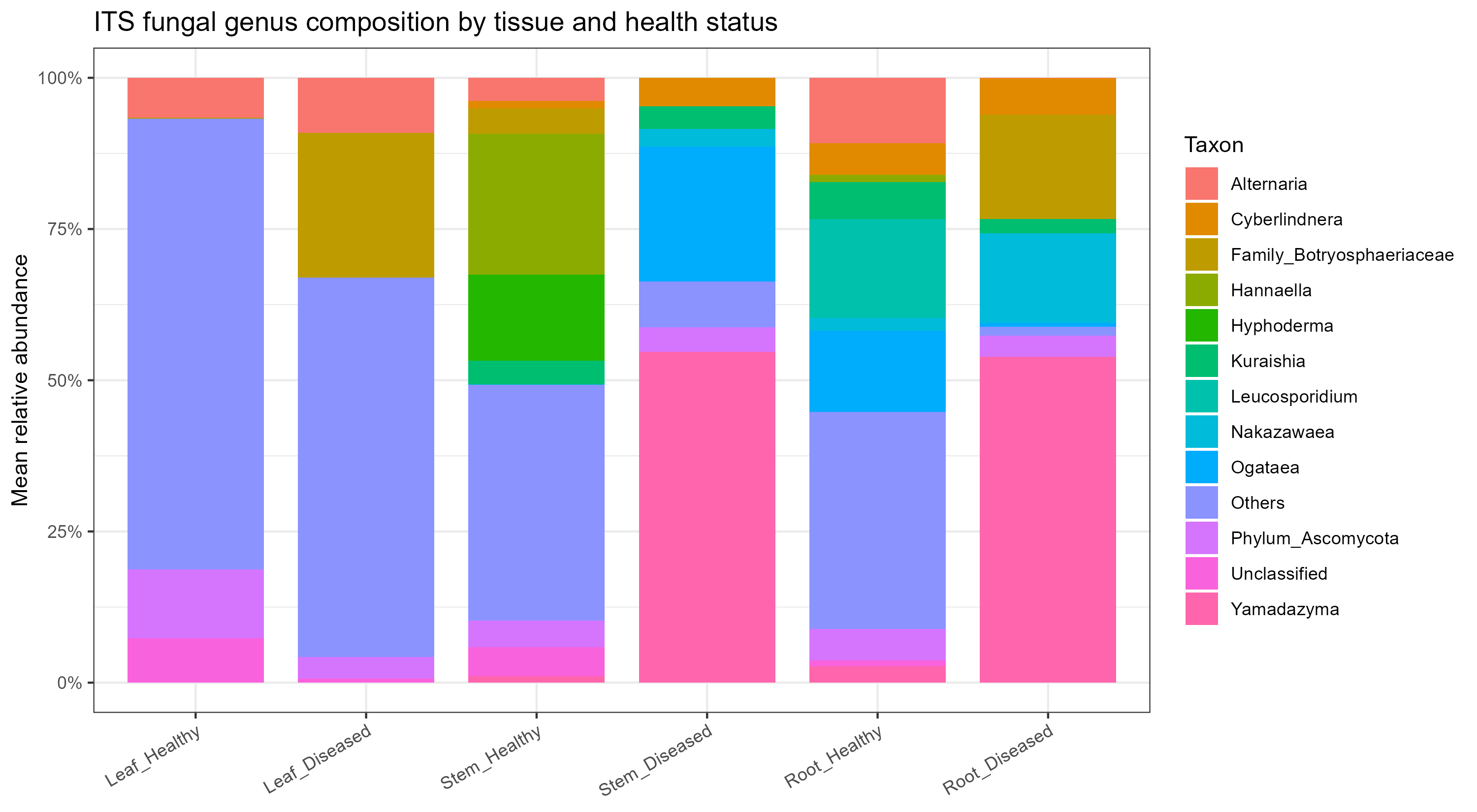

Supplement: Supplementary file 4 [file Image_4.PNG]

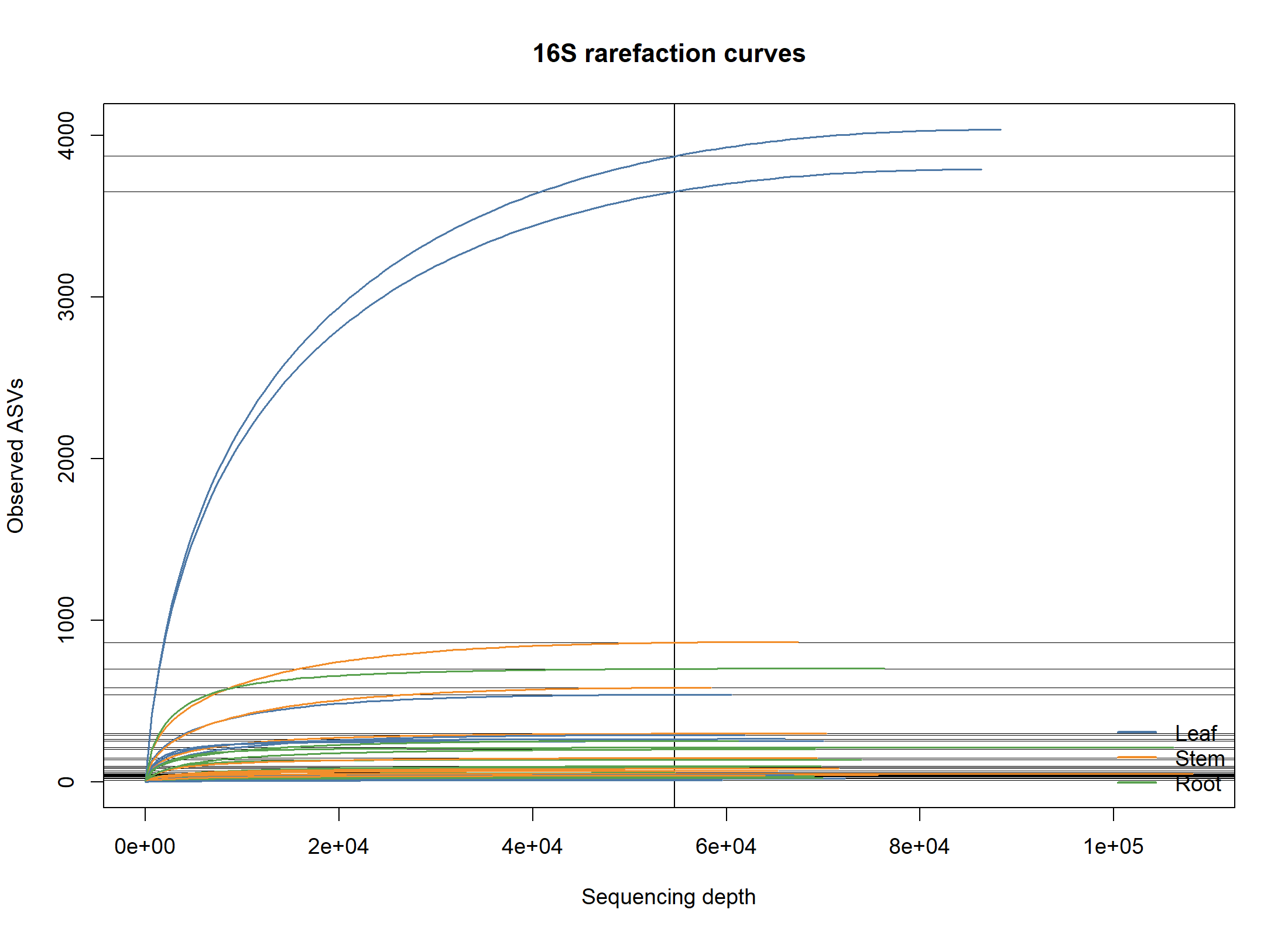

Supplement: Supplementary file 5 [file Image_5.PNG]

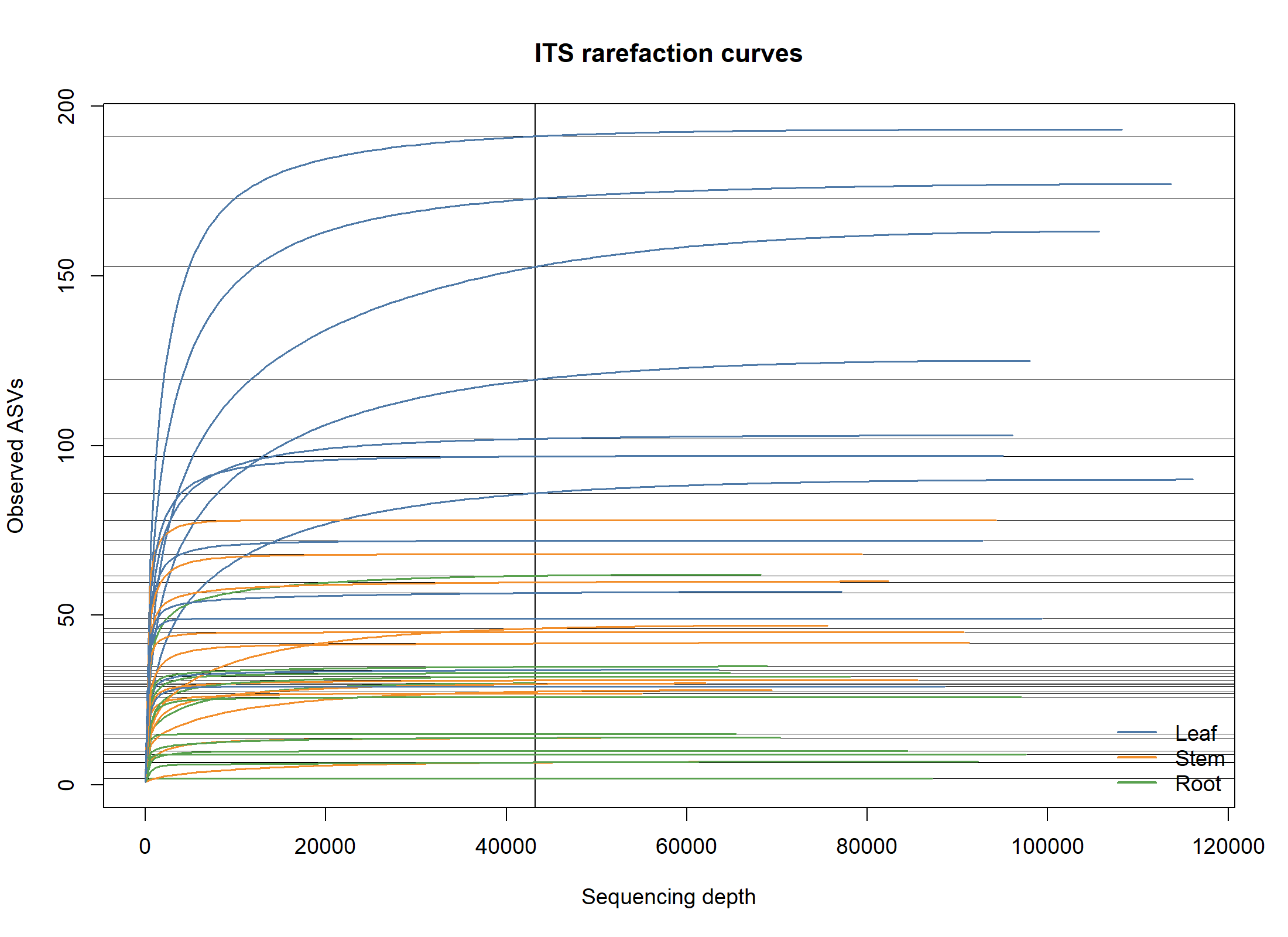

Supplement: Supplementary file 6 [file Image_6.PNG]
